# Machine Learning‐Driven Analysis of COVID‐19 Vaccination Effects on Alzheimer’s Disease Progression: A Retrospective Cohort Study

**DOI:** 10.1002/alz.095302

**Published:** 2025-01-09

**Authors:** Rifaldy Fajar, Prihantini Prihantini, Elfiany Elfiany, Sahnaz V Putri

**Affiliations:** ^1^ Yogyakarta State University, Sleman, Special Region of Yogyakarta Indonesia; ^2^ Bandung Institute of Technology, Bandung, West Java Indonesia; ^3^ BLK General Hospital, Bulukumba, South Sulawesi Indonesia; ^4^ International University Semen Indonesia, Gresik, East Java Indonesia

## Abstract

**Background:**

Recent evidence suggests a potential link between systemic inflammation induced by viral infections like COVID‐19 and the exacerbation of neurodegenerative processes in Alzheimer’s disease (AD). This study aims to explore the novel idea that COVID‐19 vaccines may offer neuroprotective effects against the progression of AD, leveraging machine learning techniques to analyze multimodal data sets.

**Method:**

We conducted a retrospective cohort study involving 10,000 participants aged 65 and older, with an initial diagnosis of early‐stage AD, from five distinct geographic locations. Participants were divided into two groups: those who received a COVID‐19 vaccine (n = 5,000) and those who did not (n = 5,000), matched for age, sex, comorbidity, and baseline cognitive function. We utilized a combination of machine learning models, including deep learning neural networks and gradient boosting machines, to analyze longitudinal data encompassing genomic profiles, clinical assessments, and neuroimaging data collected over a two‐year period. The primary outcome was the rate of cognitive decline, measured semi‐annually using the Mini‐Mental State Examination (MMSE) and the Alzheimer’s Disease Assessment Scale‐Cognitive Subscale (ADAS‐Cog).

**Result:**

The vaccinated group showed a statistically significant slower rate of cognitive decline compared to the unvaccinated group. Specifically, the annual MMSE score deterioration was 1.2 points less in the vaccinated group (95% CI: 0.8‐1.6, p<0.001). Additionally, the ADAS‐Cog progression rate was reduced by 30% (95% CI: 25%‐35%, p<0.001). The machine learning models identified a significant interaction between vaccine‐induced immune response markers and a slower progression of AD‐related neurodegenerative changes in neuroimaging data.

**Conclusion:**

Our findings suggest that COVID‐19 vaccination has a neuroprotective effect in early‐stage Alzheimer’s disease, likely due to reduced systemic and neuroinflammation. This study reveals the potential therapeutic benefits of vaccination in older adults at risk of Alzheimer’s and urges further research to understand the biological mechanisms and long‐term outcomes.

